# What do we know about elite athlete oral health?

**DOI:** 10.1038/s41415-025-8909-7

**Published:** 2026-02-27

**Authors:** Ian Needleman, Julie Gallagher, Paul Ashley

**Affiliations:** Centre for Oral Health and Performance, UCL Eastman Dental Institute, London, UK

## Abstract

**Aims** Multiple studies have identified that dental caries, periodontal diseases and erosive toothwear are common in elite athletes and possibly more prevalent than in non-athlete populations. The aims of this paper are to: 1) explore what we know about oral health in elite athletes and why we should consider athletes a priority for oral health interventions; 2) to consider the determinants of oral health and how to tackle the potential risks to athletes; and 3) to provide a summary for providers of oral health care and oral health policy, as well as athletes and sports medicine practitioners and allied professionals.

**Results** Oral diseases are common in elite athletes with some conditions, especially dental caries, likely at higher levels than the general population. There is a network of determinants at individual athlete, team and societal/policy level. Implementation of change will require user involvement, behaviour change science and an understanding of the ecosystems of elite sport. Low-cost interventions with good evidence of benefit can be implemented especially harnessing specific motivators in elite sport.

**Conclusions** The burden of oral disease in elite athletes can affect performance, health and wellbeing, and may confer a life-long shadow of treatment and disadvantage. Elite sport should be viewed as a priority for oral health improvement. Simple interventions can improve oral health in high performance sport but need to be underpinned by behaviour change science. Maintaining the oral health of athletes supports their high-level performance goals of athletes and addresses the duty of care. Furthermore, there might be the opportunity for significant oral health capital from the athletes as role models to the rest of the population.

## Key points

- Good oral health is rare in elite athletes, with dental caries, periodontal diseases and erosive tooth wear common findings.
- Many athletes will be at increased risk of oral diseases due to the use of sugars to fuel the energy demands of training, competition and recovery, as well as a number of other factors. As a result, oral health promotion may be better reframed as risk mitigation.
- Elite athletes are usually part of a multi-level ecosystem including peers, coaches, dieticians, sport and exercise medicine specialists, and federations. Therefore, all strata should be considered for involvement when developing oral health strategies.
- Carefully designed behaviour change interventions can achieve improvements in oral health behaviours in high performance sport.

## Introduction

### Elite athlete health

For every week affected by injury or illness, an athlete's odds of success in achieving their key performance goal can be reduced by 26%.^1^ Seemingly insignificant health problems can be the difference between success and failure.^2^ Therefore, even if only through the lens of performance, health promotion is highly important for elite athletes. However, the significance and value of athlete health is much broader, encompassing dimensions such as wellness, life quality, psychosocial impacts and the potential lifelong treatment shadow that could arise from ill health or injuries sustained as an athlete. Put simply, there is no health without oral health.^3^ Oral health has perhaps been slow to emerge as a key element of athlete health, mirroring the separation until relatively recently between oral health and general health. The research base is now catching up with the potential to inform on athlete health and wellbeing guidance; although, there remains much to do.

The purpose of this narrative review is to summarise:

- What do we know about the oral health of elite athletes?
- What are the determinants of elite athlete oral health?
- What are the opportunities to promote elite athlete oral health?

## Method

We searched for papers published since our systematic review^4^ using the search terms ‘Athlete' (MeSH) or ‘athlete' (title/abstract) or ‘Sports' (MeSH) or ‘sport' (title/abstract) AND ‘Oral health' (MeSH) or ‘oral health' (title/abstract) or ‘tooth' (Mesh) or ‘tooth/teeth/dental' (title/abstract) in OVID Medline up to 27 November 2024. The focus will be on adult elite athletes rather than sub-elite and recreational athletes; although, there is likely to be much that is relevant to all those active in sport.^5^ For the purposes of this paper, elite athletes are those at international level, top-tier professional leagues or tours of competition, or national level selected to represent nation.^6^ While the consequences of poor oral health will be considered briefly, the impacts on performance are the topic of another paper in this special issue, as is youth sport. The important topic of sport trauma will also be covered in separate a paper.

### What do we know about oral health in elite athletes?

It must be noted that there are some important methodological limitations that can make it difficult to draw an accurate overview when comparing studies. A striking finding is the variability in oral health levels between studies, even in apparently similar cohorts of athletes. A major component to this heterogeneity is the variability in two aspects of research. One is the lack of consistent choices of outcome measures for each health condition including the use of unvalidated outcomes.^7^ The second component is the variability in the training of examiners, with ‘no training' frequently reported,^8^ calling into question the consistency and accuracy of the measurements.

#### Dental caries

Dental caries is consistently reported to be the most common oral health condition affecting elite athletes.^9^ It can affect appearance and cause psychosocial impacts such as effects on confidence and relaxation.^10^ Caries is also associated with raised systemic inflammation,^11^ a life-long legacy of increased treatment need,^12^ dento-alveolar infection and pain. The decayed, missing, filled teeth (DMFT) index has been widely used to assess dental caries experience. The reported prevalence ranges from 15–75%.^4^ Meta-analysis on five eligible studies reported an overall caries prevalence of 46.3% (95% CI: 28.7–64.3) but with high heterogeneity (I^2^ = 93.2%). Four of the five included studies were assessed as moderate risk of bias and one as low risk.^13^ While few studies were able to make direct comparisons with non-athlete groups, this level of caries is higher than prevalence figures for similar age cohorts in many countries.^14^ Alarmingly, caries experience begins early in Academy Football.^15^

#### Periodontal diseases

Two inflammatory conditions are of interest: gingivitis, which is reversible, and periodontitis, which leads to progressive loss of the connective tissue and bone support around teeth and is irreversible. Both conditions are caused by environmental shifts in the dental biofilm (plaque) called dysbiosis.^16^ Typically, this dysbiosis occurs when daily plaque control is ineffective; although, other risk factors, such as a frequent sugar intake, favours an increase in inflammation with little change in microbial composition.^17^ In addition to the symptoms from periodontal diseases and decreased self-reported confidence and oral health-associated quality of life,^18^ both gingivitis and periodontitis can induce raised systemic inflammation,^19^ which may have consequences for athletes.

A wide range of outcome measures have been employed to assess periodontal diseases directly or indirectly, including the basic periodontal examination screening index, plaque index, bleeding on probing, gingival index, and probing depths. Overall, reported values for gingivitis prevalence range from 58–77% with a wider range in values for periodontitis but most commonly 15–41%.^4^^,^^8^ In a young population in Germany, the prevalence of deeper pockets >4 mm suggestive of periodontitis (maximum Periodontitis Severity Index score 3) was 40% in competitive athletes versus 12% amateur athletes (*p* <0.0001).^20^

#### Erosive tooth wear

Tooth wear is defined as the cumulative surface loss of mineralised tooth substance due to physical or chemo-physical processes (dental erosion, attrition, abrasion) and is not considered to be the result of dental caries, resorption, or trauma.^21^ Within this group of processes, erosive tooth wear (ETW) has received most attention in elite sport. It is caused by exposure to acids not derived from oral bacteria, either endogenous (gastric fluids from bulimia or reflux) or exogenous, e.g., from diet (including sports drinks), environment and/or drugs.^21^ It can be difficult to distinguish tooth wear clinically between the different processes. The prevalence of disordered eating in elite sport varies widely but is consistently higher in aesthetic and weight-critical sports. The overall self-reported prevalence of disordered eating in elite athletes is 19.2% (95% CI: 17.04–21.6%, I^2^ = 97.4) and higher than non-athlete populations.^22^ Therefore, early identification of ETW in athletes is important in screening for disordered eating which can have severe health consequences if not managed appropriately.

Although several approaches to assessing ETW were employed in these studies, most used standardised methods.^23^ The range of prevalence of ETW in elite athletes is 36–85%,^4^ including three studies and 434 athletes. A more recent meta-analysis included nine studies and involving,1,550 elite and non-elite athletes estimated the prevalence at 47.07% (95% CI: 24.03–70.80) and with very high heterogeneity (I^2^ = 99.01). Of the studies, three were at moderate risk of bias and six at low risk, with one study comparing prevalence rates in athletes with similar age youth population data reporting values twice as high for the athletes (59% versus 20–25%).^24^^,^^25^

#### Other oral health conditions/problems

Infections around wisdom teeth (pericoronitis) can cause significant morbidity and affect quality of life. They are of particular relevance as their peak age of incidence coincides with the typical age of elite athletes.^26^ Pericoronitis has been evaluated in only very few studies. Reported values for prevalence including data on pericoronitis or the presence of impacted wisdom teeth which might not have active infection ranged from 4.5–39% of studies.^4^

It is useful to capture more severe manifestations of oral infection, including those resulting from untreated caries or periodontitis. As a result, the PUFA index was developed and validated.^27^ The components are P: pulpal involvement with open pulp chamber, U: ulceration from trauma resulting from sharp tooth edges, F: fistula with a sinus draining pus and A: abscess with pus related to a tooth with pulpal involvement. When a large cohort of elite athletes were examined (n = 352) 3.4% had at least one PUFA finding.^14^

Temporomandibular disorders (TMD) are a range of conditions with multifactorial complex pathophysiology, which are common up to the age of 40 years.^28^ Some of these conditions can also cause significant morbidity through pain and difficulty eating. Diagnostic criteria have been developed with validated tools recommended.^29^ However, assessment of the studies in athletes is complicated by some evaluating symptoms and others ‘signs' or habits (such as tooth grinding) regarded as manifesting an underlying TMD condition. While gaining a true picture is therefore problematic, the overall reported prevalence of TMD in elite athletes would appear to be low at <7% athletes.^4^

#### Summary

Overall, dental caries, periodontal diseases and ETW are common in elite athletes. Although showing marked variability between studies, the prevalence of caries and ETW appear to be higher than comparable age non-athlete cohorts. It would also appear that disadvantage starts early with Football Academy players already showing compromised oral health.^15^

### What are the determinants of elite athlete oral health?

#### The broad spectrum of health determinants

**Fig. 1:**
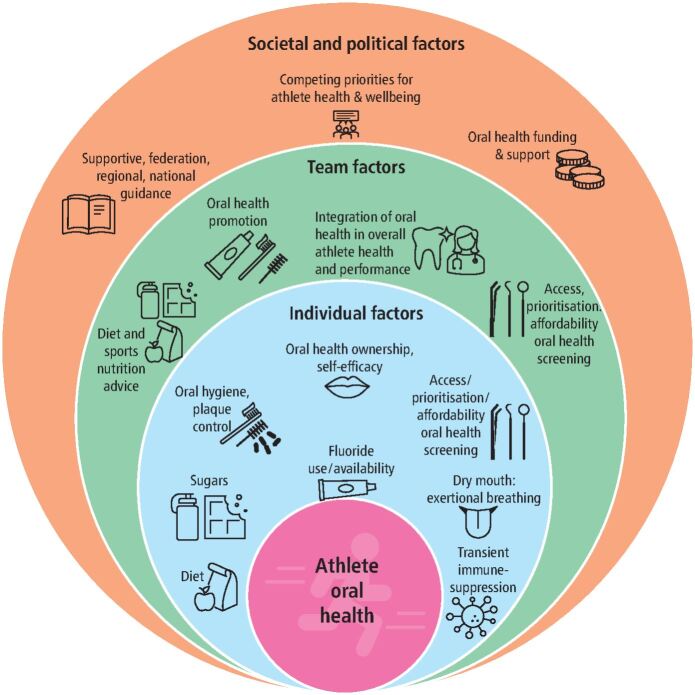
A suggested model of the multi-level determinants of elite athlete oral health. The athlete is at the centre of the model with more upstream determinants at team and societal/political levels (based originally on Dijkstra *et al.*)^30^

This paper will only consider a limited summary of the chief determinants of oral health since more comprehensive reviews are available.^8^^,^^9^ The determinants of athlete oral health should be considered within the overall framework of determinants of oral health for any individual. This is an important consideration as much is already known and characterised outside the specifics of sport. Figure 1 presents a suggested model of the multi-level determinants of elite athlete oral health. The athlete is at the centre of the model with more upstream determinants at team and societal/political levels. The ecosystem will contain important determinants for their oral health, therefore providing significant opportunities for health improvement.^30^ Focusing interventions only on factors further downstream (such as diet and oral health behaviours), although important, is likely to have a less sustained effect than measures implemented further upstream and could increase health disparity.^31^^,^^32^^,^^33^ Athletes are usually part (although hopefully at the centre) of an ecosystem or community. A recent review of the barriers and facilitators for integration of oral health into primary care^34^ shares many similarities with the high-performance sport model ^30^ and provides further structure for planning.

#### Athlete and team knowledge, agency and ownership of oral health

Oral diseases are common in athletes; however, when surveyed, they generally demonstrate good knowledge of the causes of the conditions as well as reporting appropriate oral health behaviours, typically better than might be expected in non-athlete populations.^35^ This dissonance between actual athlete oral health and their reported behaviours requires consideration. Unfortunately, very little is known about what athlete support members (coaches, nutritionists, performance directors, etc.) know, believe and promote about oral health but this is likely to be highly influential to athletes and might help to unravel part of this contradiction. Stakeholder engagement is likely to be critical in achieving change.^36^ Guidance aimed at athletes, teams and organisations does exist, but the level of reach is unknown.^37^^,^^38^^,^^39^^,^^40^

#### Nutrition and dental caries

Carbohydrates, particularly sugars, are used by athletes to fuel training, competition and recovery. In relation to oral health, an increase in the amount and frequency of consumption of sugar is a key cause of dental caries.^41^ Endurance athletes often use sugars at levels of 90 g/h or more for considerable periods, and increasingly as mouthrinses for rapid energy availability.^42^ As a result of the dental biofilm metabolism of the sugars, plaque pH may be depressed for long periods of time favouring the development of dental caries. While salivary buffering and dilution are important homeostatic mechanisms to restore equilibrium in the mouth, high airflow and possibly stress can reduce saliva availability, therefore enhancing the persistence of a low plaque biofilm pH.

#### Nutrition and inflammation

The influence of a high sugar intake extends beyond dental caries. While the quantity of the dental biofilm does not appear to increase with frequent sugar intake, there is a greater inflammatory response from the periodontal tissues,^43^ i.e., gingivitis and potentially therefore a greater risk of periodontitis.

#### Effective oral hygiene

Maintaining oral health depends on regular, effective disruption/removal of the dental biofilm (plaque control or oral hygiene). Currently, the advice is a minimum of twice daily toothbrushing for at least two minutes and interdental plaque control at least once per day.^41^ Interestingly, when surveyed, athletes know much of this guidance and report adhering to it. However, high levels of gingivitis found in most surveys of athletes demonstrate that there is an implementation gap between knowledge and practice.^14^^,^^35^^,^^36^

#### Fluoride

To protect against dental caries, the current recommendation for all adults is to use a toothpaste with at least 1,450 ppm fluoride. However, for those at high risk of caries (as demonstrated by many athletes), higher levels of fluoride are recommended, including toothpastes with up to 5,000 ppm used twice per day.^41^ To maintain the fluoride levels, the toothpaste should not be rinsed away leading to the advice, ‘spit, don't rinse' following brushing. To increase availability further, the athlete at high risk of dental caries should also rinse at another time of day with a mouthwash containing 0.05% sodium fluoride.

#### Access, prioritisation and funding

Other determinants of oral health for an athlete will include their access, prioritisation (motivation) and indeed affordability of oral healthcare, and these factors could operate at all levels of the ecosystem, i.e., for the individual, team and federation. In most countries and sport federations, oral healthcare is not provided or subsidised for athletes. These factors are also likely to be markedly affected by the periodisation of athlete training and competition during the year, hence the importance of oral health screening in the ‘pre-season' whenever possible. Therefore, individuals will need to find a dentist which is likely to act as a significant barrier for many. Athlete health and wellbeing have many competing priorities, including COVID-19 (in recent years), mental health and head injury being just a few areas of focus that have dominated research and guidance. Oral health may not yet have made its case sufficiently for consideration in high level athlete health agendas.

#### Summary

The determinants of athlete oral health should consider not only those under direct control of the athlete but the wider ecosystem of support around the athlete. For many athletes, high sugar frequency is needed to ensure optimal fuelling and recovery. Promoting oral health in elite athletes might then be better considered as risk mitigation. Athletes appear to understand both the causes of oral diseases and the recommended oral health behaviours. Therefore, enhancing athlete knowledge alone is unlikely to influence oral disease levels.

### What are the opportunities to promote elite athlete oral health?

The discussion of determinants might be interpreted as posing too great a series of challenges to improve athlete oral health. However, this would be an inaccurate conclusion. Instead, the specific context of elite sport needs to be understood to make progress. There are two important strengths that highlight the potential for change in this unique population group. The first is that athletes are exceptionally good at following structured programmes, if they and their ecosystem of support embrace it. The second is that there is a wealth of low-cost interventions with good evidence of efficacy to promote oral health and prevent oral diseases. Bringing these two elements together is likely to achieve progress.^5^

#### Health, risk and motivation

In terms of oral health *per se,* there is no reason to believe that athletes would be more or less interested in oral health than non-athletes.^35^ However, a key difference might be their consideration of risk. Elite athletes are highly target-driven to the extent that accepting risk, including in health behaviours, may be less important than non-athlete populations.^44^ Therefore, interventions purely based on education about risk of oral diseases may not be effective in changing behaviour. However, behaviour change science can help to understand how to overcome these limitations and to design successful interventions.^45^

### Can oral health behaviours be changed in high performance sport?

When working with athletes and their team members in GB Rowing, GB Cycling and a professional premiership rugby team, we identified two factors likely to motivate the athletes to change their behaviour:

- Oral health to prevent systemic inflammation
- Oral health for appearance.

These motivators were included in a feasibility study to test whether it was possible to improve oral health in high performance sport.^36^

**Fig. 2:**
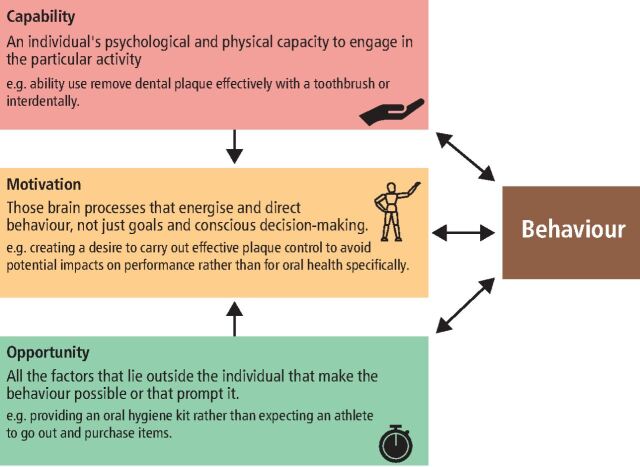
The COM-B framework to help understand behaviour (modified) with examples related to plaque control^43^

Motivation is essential for behaviour change; however, it is interdependent upon people having the opportunity and capability (knowledge and skills) to effect real change (Fig. 2). We addressed ‘opportunity' by providing an oral health kit, containing the recommended elements for effective oral hygiene to each athlete. The oral health behaviours encouraged were:

- Toothbrushing for at least two minutes twice per day (spitting but not rinsing after toothbrushing)
- To increase fluoride availability with a high fluoride toothpaste (2,500 ppm)
- Interdental plaque control with floss wands.

‘Capability' was addressed by providing a 10-minute intervention for athletes and their support team together plus three instructional 90-second videos, aiming to build motivation, knowledge and skills for all. Secondly, for athletes alone, a personal oral health screening, brief personalised advice based on screening and provision of the oral health kit. Importantly, the intervention was designed to address these different levels of the ecosystem.

The preventive message conveyed to the athletes and their support team was based on that in the toolkit *Delivering Better Oral Health 2021*^41^ for ‘adults giving concern because of dental caries' risk. We had previously identified these behaviours as potentially implementable in a study of a large cohort of elite athletes.^35^ Furthermore, we communicated the routine for the behaviours in terms of athlete drills based around a standard training day. Athletes were followed up at 4–8 weeks and 12–18 weeks.

At 12–18 weeks, a key measure of success was that 55/62 (89%) of athletes completed all visits despite their busy schedule. All three primary outcomes – oral health knowledge, oral health behaviours and self-reported performance impacts – showed statistically significant improvements. As a check back at the end of the study completed by all 55 athletes, the highest rated motivator for change was wishing to minimise inflammation elsewhere in the body. Other studies are needed to explore different methods of health behaviour change with sufficient follow-up to assess oral health outcomes themselves.

Another approach that shows promise for behaviour change is gamification. In relation to health behaviours, gamification has many similarities with the tracking, goal setting and app-based monitoring already routinely employed by athletes and their support teams. A recent scoping review of gamification in children and adolescents of 15 studies reported particular promise in relation to enhancing oral health literacy and behaviours.^46^

### Other health behaviours to mitigate risk

The need for sugar to fuel training, competition and recovery cannot be escaped. Therefore, oral health promotion is probably more meaningfully reframed as risk mitigation in elite sport. The evidence for the benefit of such approaches is lacking and needs to be developed. However, there are a number of options that would seem sensible to adopt, at low financial cost and at low risk of harm and readers are recommended to a recent infographic:^38^

- For all athletes, a food first approach to diet emphasising whole foods rather than supplements whenever possible to gain best advantage of the complexity of micronutrients/macronutrients
- Diluting sugar intakes in the mouth by using a two-bottle approach, i.e., water following use of a sports drinks (or gel) with sugar
- Using water or milk to rehydrate rather than sports drinks with sugars
- Regular oral health screening should be standard for elite athletes.

Oral health screening should be routine for elite athletes. The evidence highlights that many athletes do not attend regularly for screening with cost and access to care frequently cited as barriers by them. The International Olympic Committee recommended that periodic health evaluation for all athletes included assessment of oral health;^47^ although, this was mainly limited to dental caries. Following our evaluation of professional football, together with Manchester United and West Ham FC, we also called for action as the evidence pointed to a stronger need for oral health screening than many of the standard musculoskeletal screening that was routinely employed.^48^ Frequency of screening will depend on risk. In view of the levels of oral disease, it would seem sensible to implement initially twice-yearly screening and to revise the frequency as the individual athlete's risk becomes clearer. At least one screening should be in the low season or equivalent to allow any treatment that is identified to be carried out with minimal disruption to the athlete. However, it is critical that the opportunity is taken at the time of screening to ‘coach' the athlete with an individualised preventive programme based on their risk. Self-reported oral health could prove an additional measure to supplement clinical screening programmes similar to other athlete health monitoring including sleep quality.^36^^,^^49^ Further research will be needed to determine the utility of such an approach.

## Conclusions

The evidence points to a significant burden of oral disease in elite athletes. Not only is this likely to affect their performance, health and wellbeing, but may confer a life-long shadow of treatment and disadvantage. Elite sport should be viewed as a priority for oral health improvement. By maintaining oral health, not only are we supporting the high-level performance goals of athletes but also our duty of care will help to set them up for the future once they retire from elite sport. Furthermore, there might be the opportunity for significant oral health capital from the athletes as role models to the rest of the population.

It has been recognised for many years that the most disadvantaged bear both the greatest burden of disease and the greatest difficulty in accessing care.^50^ Interventions to maintain oral health, particularly in populations considered ‘at risk' are well-known, effective and low-cost. Implementing them into high performance sport can be achieved but requires an understanding of their unique ecosystems and psychology. Finally, for future research, we recommend including validated core outcomes to better allow comparison of oral health in different studies and a better understanding of the determinants of oral health and the outcomes of health-improvement interventions.^7^
